# The DEAD/DEAH box helicase, DDX11, is essential for the survival of advanced melanomas

**DOI:** 10.1186/1476-4598-11-82

**Published:** 2012-11-01

**Authors:** Chitralekha Bhattacharya, Xiaolei Wang, Dorothea Becker

**Affiliations:** 1 Department of Pathology, University of Pittsburgh, HCC 1.46, 5117 Centre Avenue, Pittsburgh, PA 15213, USA; 2 Present address: Department of Medicine, University of Pittsburgh, HCC 1.18, 5117 Centre Avenue, Pittsburgh, PA, 15213, USA; 3 Molecular Imaging Center, Department of Radiology, University of Pittsburgh, 100 Technology Drive, Pittsburgh, PA, 15219, USA

**Keywords:** Melanoma, DDX11, Chromosome segregation defects, Inhibition of proliferation, Apoptosis

## Abstract

**Background:**

Despite continuous efforts to identify genes that are pivotal regulators of advanced melanoma and closely related to it, to determine which of these genes have to be blocked in their function to keep this highly aggressive disease in check, it is far from clear which molecular pathway(s) and specific genes therein, is the Achilles’ heel of primary and metastatic melanoma. In this report, we present data, which document that the DEAD-box helicase DDX11, which is required for sister chromatid cohesion, is a crucial gatekeeper for melanoma cell survival.

**Methods:**

Performing immunohistochemistry and immunoblot analysis, we determined expression of DDX11 in melanoma tissues and cell lines. Following transfection of melanoma cells with a DDX11-specific siRNA, we conducted a qPCR analysis to determine downregulation of DDX11 in the transfected melanoma cells. In subsequent studies, which focused upon an analysis of fluorescently labeled as well as Giesma-stained chromosome spreads, a proliferation analysis and apoptosis assays, we determined the impact of suppressing DDX11 expression on melanoma cells representing advanced melanoma.

**Result:**

The findings of the study presented herein document that DDX11 is upregulated with progression from noninvasive to invasive melanoma, and that it is expressed at high levels in advanced melanoma. Furthermore, and equally important, we demonstrate that blocking the expression of DDX11 leads not only to inhibition of melanoma cell proliferation and severe defects in chromosome segregation, but also drives melanoma cells rapidly into massive apoptosis.

**Conclusion:**

To date, little is known as to whether helicases play a role in melanoma development and specifically, in the progression from early to advanced melanoma. In this report, we show that the helicase DDX11 is expressed at high levels in primary and metastatic melanoma, and that interfering with its expression leads to severe chromosome segregation defects, telomere shortening, and massive melanoma cell apoptosis. These findings suggest that DDX11 could be an important candidate for molecular targeted therapy for advanced melanoma.

## Background

Molecular therapy targeting the BRAF^V600E^ mutation and anti-CTLA-4 antibody-based immunotherapy have shown some benefit for patients with advanced melanoma. However, in light of the fact that advanced melanoma, rapidly and very aggressively, mounts resistance to BRAF small-molecule inhibitor treatment; anti-CTLA-4 immunotherapy has efficacy in less than 10-20% of melanoma patients; and advanced melanomas harbor neither a large number of gene fusions or chimeric transcripts [1] nor with the exception of the BRAF^V600E^ mutation, a high rate of mutations [2,3], the pertinent challenge regarding this disease continues to be the identification of genes that are upregulated to high levels in advanced melanoma, and when inhibited in their function, cause primary and metastatic melanoma cells to undergo apoptosis.

In line with our long-term effort to identify genes that are upregulated with progression from early to advanced melanoma, we recently profiled archived, formalin-fixed paraffin-embedded tissue samples representing early as well as advanced melanoma on DASL BeadChip arrays. The data from this whole-genome expression analysis revealed that one of the identified genes, upregulated to substantial levels with progression from noninvasive melanoma in situ > to invasive radial growth phase melanoma > primary melanoma > metastatic melanoma is the gene DDX11, which has never before been associated with melanoma.

First isolated as the human homologue of the yeast CHL1 gene [4,5], DDX11 (alias ChlR1) is a member of the DEAD/DEAH box family of helicases, which comprises more than 40 members. Sharing sequence similarity with the FANCJ helicase and the DEAH-box helicases, XPD and RTEL [6], DDX11 is essential for the cohesion of chromosome arms and centromeres and when depleted, mitotic failure occurs due to replicated chromosomes failing to segregate after prometaphase arrest [7]. More recently, biallelic mutations in DDX11 have been identified as the cause of the Warsaw breakage syndrome cohesinopathy, which among other clinical manifestations is associated with abnormal skin pigmentation [8].

The findings of our study, summarized herein, demonstrate that DDX11 is expressed at high levels in primary and metastatic melanomas, but not in melanocytes of normal skin, atypical nevi, or melanoma in situ, and that suppressing DDX11 expression in advanced melanomas leads to severe defects in chromosome segregation, and with potential relevance to therapeutic intervention, inhibition of melanoma cell proliferation and rapid melanoma cell death.

## Results

### Status and pattern of DDX11 expression in normal skin, nevus and melanoma tissues, and melanoma cell lines

To identify genes that are upregulated with progression from noninvasive melanoma in situ (MIS) to radial growth phase (RGP) melanoma, which is the first stage of invasive melanoma, we recently subjected RNAs isolated from archived, formalin-fixed paraffin-embedded (FFPE) tissue samples representing these two stages of early melanoma development to whole-genome DASL HT BeadChip arrays. Analysis of the not background subtracted, but quantile normalized data of this whole-genome expression profiling study, which as a subsequent step, included a stringent Ingenuity Pathway (IPA) core analysis, revealed that DDX11, a gene never before associated with melanoma, was upregulated 8-fold in RGP melanoma compared with MIS.

DDX11 (alias ChlR1), one of the members of the DEAD/DEAH box family of helicases, is required for the cohesion of chromosome arms and centromeres [5,7,9] and thus, plays an important role in maintaining genome stability. In view of the fact that, to date, little is known regarding the role of helicases in melanoma and likewise, regarding genes that have pertinent functions in maintaining chromosome transmission fidelity and genome stability in melanoma cells, we deemed it important to characterize the function(s) of DDX11 in melanoma. As a first step towards this goal, we performed immunohistochemistry analysis of tissues as well as immunoblot analysis of cell lines to determine the status and pattern of DDX11 expression in normal skin, nevus and melanoma tissues, and in cell lines representing primary melanoma in the vertical growth phase (VGP melanoma) and melanoma in the metastatic growth phase (MGP melanoma).

**Figure 1:**
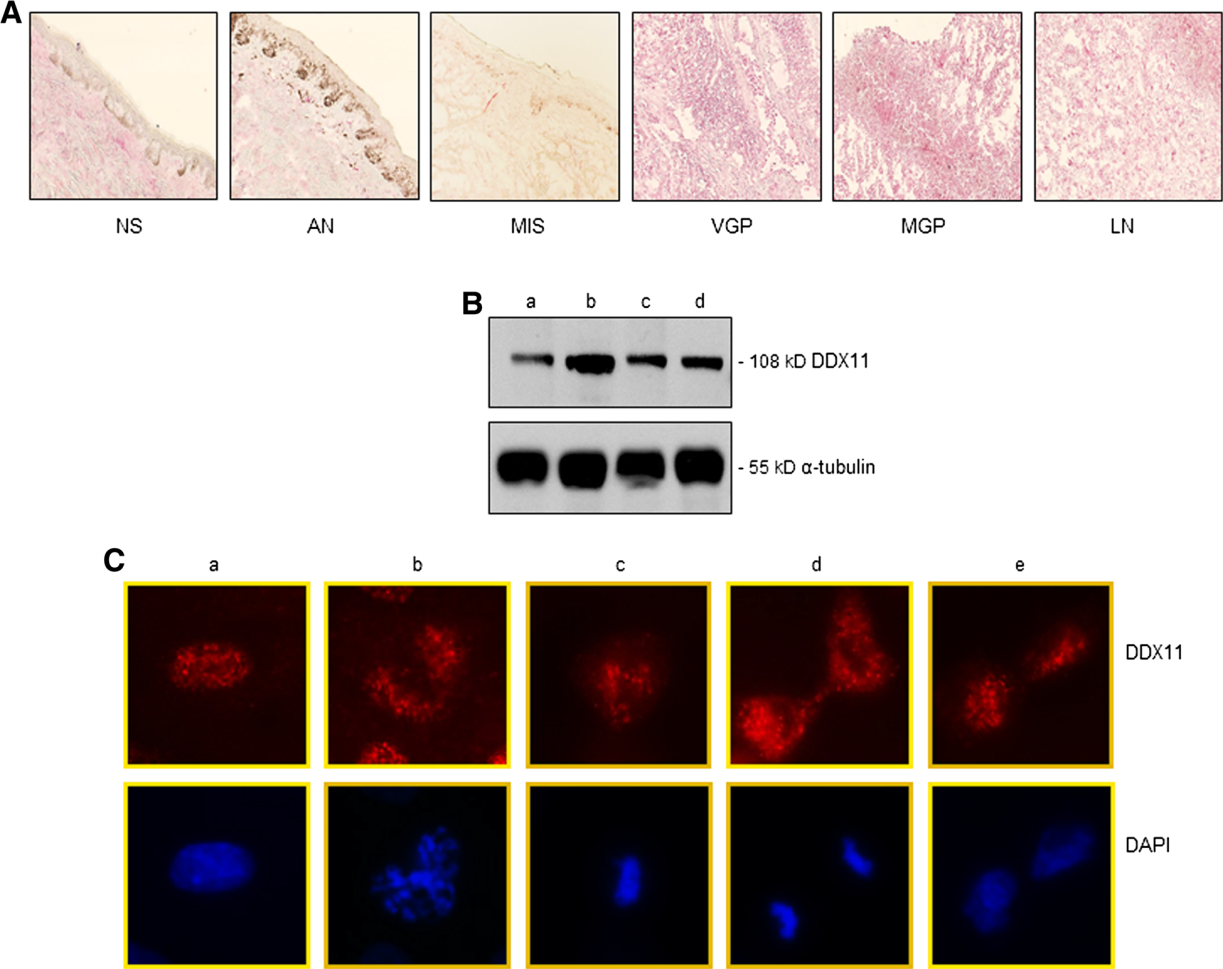
**DDX11 expression in normal skin, nevi, and melanoma tissues and cell lines.** (**A**) Cryopreserved tissue sections, representing normal human skin (NS), atypical nevus (AN), melanoma in situ (MIS), VGP melanoma (VGP), MGP melanoma (MGP), and melanoma-infiltrated lymph node (LN), were stained with an antibody to human DDX11 and counterstained with hematoxylin. Images were captured at 10X magnification. (**B**) Immunoblot analysis of VGP melanoma cells WM983-A (a), and MGP melanoma cells WM983-B (b), WM852 (c), and WM1158 (d) probed with DDX11 antibody. For loading control, the immunoblot was probed with an antibody to α-tubulin. (**C**) Immunofluorescence analysis of DDX11 expression in MGP melanoma cells, WM1158, during interphase (a), prophase (b), metaphase (c), telophase (d) and late telophase (e). The melanoma cells were probed with an antibody to human DDX11 (pseudocolored red), and their nuclei (pseudocolored blue) counterstained with fluorescent DAPI.

Immunohistochemistry analysis of cryopreserved tissue sections, prepared from normal skin, atypical nevi, which are the precursors and risk markers of melanoma, MIS, and advanced melanomas, with an antibody to human DDX11 revealed strong expression of DDX11 in advanced melanoma comprised of VGP and MGP melanomas and melanoma-infiltrated lymph nodes (LN) (Figure 1A). In contrast, DDX11 expression was not detected in epidermal melanocytes of normal skin (NS) (Figure 1A) - a finding that is in agreement with the data of a previous study, which showed that DDX11 is expressed at extremely low levels in normal human skin [4]. Likwise, neither atypical nevocytes (AN) (Figure 1A), which are clusters of melanocytes nor MIS cells showed expression of DDX11 (Figure 1A). Similar to the strong expression of DDX11 detected in tissues representing advanced melanoma, subsequent immunoblot analysis revealed that DDX11 is also strongly expressed in cell lines representing VGP (Figure 1B, lane a) and MGP melanoma (Figure 1B, lanes b-d). We did not include in the immunoblot analysis, protein lysates representing atypical nevocytes, MIS, or RGP melanoma cells, because none of these cells can be propagated in vitro.

Since it been reported that DDX11 has a dynamic localization during mitosis [7], we performed in addition to the immunohistochemistry and immunoblot analyses, a DDX11-based immunofluorescence analysis of WM1158 MGP melanoma cells, which as shown in Figure 1B, lane d, exhibited clearly detectable expression of the 108 kD DDX11 protein. Not pretreated with any agent to enrich for cells in mitosis, staining of WM1158 MGP melanoma cells with the DDX11 antibody served to show expression and localization of DDX11 during interphase (Figure 1C, lane a) and the subsequent stages of mitosis (Figure 1C, lanes b-e).

### Downregulation of DDX11 expression severely impairs the morphology of melanoma cells

**Figure 2:**
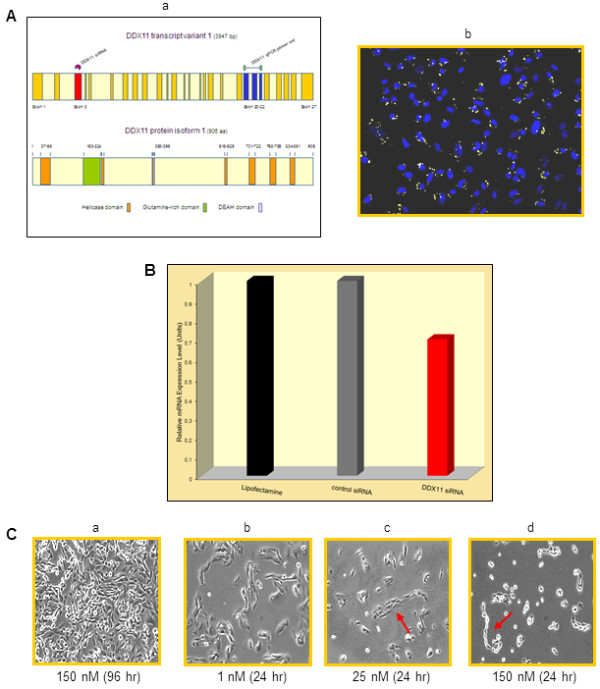
**Analysis of DDX11 siRNA-transfected melanoma cells.** (**A**, panel a) Schematic presentation of human DDX11 transcript variant 1, and protein isoform 1. The red-colored symbol above exon 3 in transcript 1 marks the position of the DDX11 siRNA, and the green-colored bar symbol above exons 20–22, indicates the location of the DDX11 qPCR primers used in the qPCR analysis. (**A**, panel b) Immunofluorescence analysis of WM1158 MGP melanoma cells at 24 hr following transfection with 5 nM of Cy5-conjugated DDX11 siRNA (pseudocolored yellow). Nuclei (pseudocolored blue) were counterstained with fluorescent DAPI. (**B**) qPCR analysis of WM1158 MGP melanoma cells that had received only Lipofectamine 2000 (black-colored bar), or were transfected with 25 nM of control siRNAs (grey-colored bar) or DDX11 siRNA (red-colored bar) for 48 hr. (**C**) Phase-contrast images, captured at 10X magnification, depicting the morphology WM1158 MGP melanoma cells following transfection with 150 nM of control siRNAs for 96 hr (a) or with 1 nM (b), 25 nM (c) or 150 nM (d) of DDX11 siRNA for 24 hr. The red-colored arrow in the phase-contrast images, shown in panels c and d, points to the chain-like morphology of melanoma cells that did not separate.

Alternative splicing of the evolutionary highly conserved human DDX11 gene, located on chromosome 12p11, which is not a chromosomal locus that is altered in advanced melanomas, yields several transcript variants of slightly different length. Depicted in Figure 2A, panel a, are schematic presentations of transcript variant 1 and protein isoform 1 of human DDX11. To determine whether inhibiting expression of DDX11 in human melanoma cells would interfere with the cells’ proliferation and possibly some of their other biologic characteristics, we used a DDX11-specific siRNA that targeting a 25 bp region in exon 3 of human DDX11 (Figure 2A, panel a), was previously reported to downregulate human DDX11 [10]. To ascertain effective uptake of this specific siRNA, we transfected WM1158 MGP melanoma cells with 5 nM of the DDX11 siRNA that we had conjugated to the cyanine dye, Cy5. As shown in Figure 2A, panel b, imaging analysis, performed 24 hr after transfection, did reveal the presence of the DDX11 siRNA in these melanoma cells.

To assess whether following transfection of melanoma cells with the DDX11 siRNA, DDX11 mRNA would be downregulated, we transfected WM1158 MGP melanoma cells with 25 nM of the DDX11 siRNA not conjugated to Cy5, and serving as control, with 25 nM of a pool of siRNAs comprised of four non-targeting siRNAs. Using a pair of qPCR primers spanning exons 20–22 of human DDX11 (Figure 2A, panel a), we performed qPCR analysis, which as depicted for the 48 hr time point post transfection (Figure 2B), revealed that expression of DDX11 was decreased when compared with WM1158 MGP melanoma cells that had received only the siRNA delivery vehicle Lipofectamine 2000, or the pool of the control siRNAs.

The most prominent phenotypic change we observed during this series of experiments was that compared with control siRNA transfected cells (Figure 2C, panel a), melanoma cells that had been transfected with the DDX11 siRNA, exhibited a rapid and dramatic alteration in their morphology. Specifically, we found that as shown in Figure 2C, panels b-d, transfection of the DDX11 siRNA caused melanoma cells to lose cell-cell contact in as little as 24 hr, and at doses of 25 nM as well as 150 nM of DDX11 siRNA, a significant number of the transfected melanoma cells exhibited a tightly held together chain-like morphology (Figure 2C, panels c and d).

### Inhibition of DDX11 expression leads to chromosome segregation defects in melanoma cells

**Figure 3:**
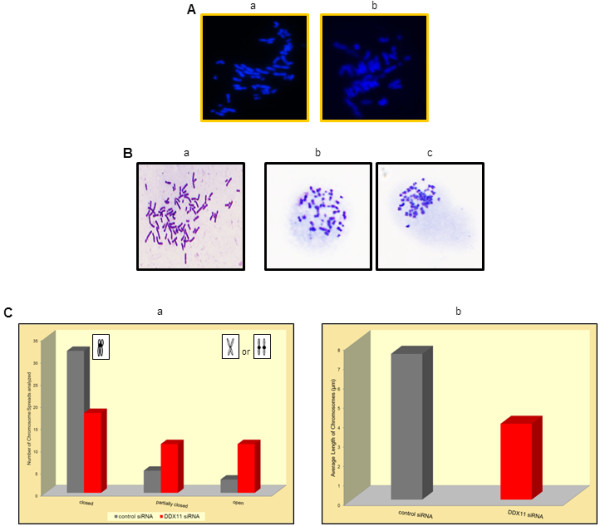
**Inhibition of DDX11 expression in melanoma cells leads to chromosome segregation defects.** (**A**) Immunofluorescence analysis of fluorescent DAPI-stained chromosome spreads (40X magnification) of WM1158 MGP melanoma cells transfected for 24 hr with 75 nM of control siRNA (a) or 75 nM of DDX11 siRNA (b). (**B**) Images of Giemsa-stained chromosome spreads of WM1158 MGP melanoma cells transfected for 24 hr with 75 nM of control siRNAs (a) or 75 nM of DDX11 siRNA (b and c). (**C**) Giemsa-stained chromosome spreads of WM1158 MGP melanoma cells transfected for 24 hr with 75 nM of control siRNAs (grey-colored bars) or 75 nM of DDX11 siRNA (red-colored bars) were analyzed for chromosomes with closed, partially closed, or open/separated arms (panel a) as well as average length of chromosomes (panel b).

To gain insights into the cause(s) of the DDX11 siRNA-induced morphologic changes, we transfected melanoma cells with the DDX11 siRNA and 24 hr later prepared chromosome spreads that were stained with either fluorescent DAPI or Giemsa solution. Compared with the DAPI-stained chromosome spreads of melanoma cells that had been transfected for 24 hr with the control siRNA pool (Figure 3A, panel a), the DAPI-stained chromosome spreads of DDX11 siRNA-transfected melanoma cells (Figure 3A, panel b) revealed a pattern of tightly condensed chromosomes. This difference in chromosome abnormality became even more apparent when the chromosome spreads of control siRNA (Figure 3B, panel a) and DDX11 siRNA-transfected melanoma cells (Figure 3B, panels b and c) were stained with Giemsa.

Since it has been reported that DDX11 plays an important role in maintaining fidelity of chromosome arm cohesion prior to mitosis [11], we next determined in a total of 40 chromosome spreads prepared from the DDX11 siRNA-transfected and likewise, the control siRNA-transfected melanoma cells, the number of chromosomes that had closed, partially closed, or open/separated arms. As displayed in Figure 3C, panel a, compared with the control, the DDX11 siRNA-transfected melanoma cells exhibited approximately 50% fewer chromosomes with closed arms, and about 50-60% more chromosomes with partially closed or open/separated arms. Furthermore, and even more apparent was that the average length of the chromosomes of the DDX11 siRNA-transfected melanoma cells was significantly shorter than the length of the chromosomes of the control siRNA-transfected melanoma cells (Figure 3C, panel b).

### Suppression of DDX11 expression severely impairs melanoma cell proliferation and causes massive melanoma cell apoptosis

**Figure 4:**
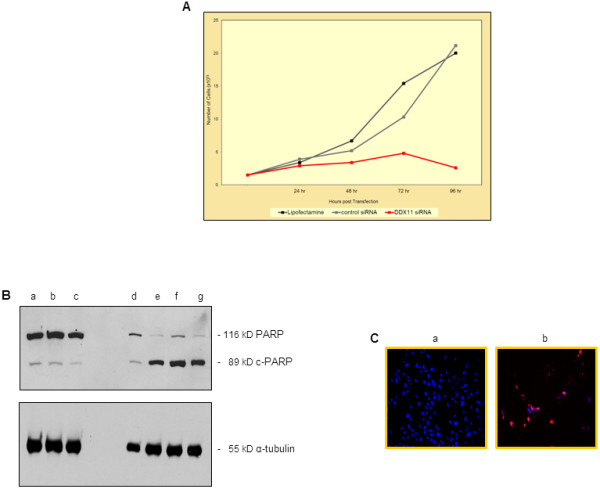
**Suppressing DDX11 expression in melanoma cells leads to inhibition of melanoma cell proliferation and massive melanoma cell apoptosis. ***(***A**) Proliferation of WM1158 MGP melanoma cells that received only Lipofectamine 2000 (black-colored line) or were transfected with 25 nM of control siRNAs (grey-colored line) or 25 nM of DDX11 siRNA (red-colored line). Depicted at each time point following siRNA transfection is the mean of duplicate samples analyzed. (**B**) Immunoblot analysis of WM1158 MGP melanoma cells, transfected with 25 nM of DDX11 siRNA for 24 hr (d), 48 hr (e), 72 hr (f) or 96 hr (g), were probed with an antibody to c-PARP. Protein lysates prepared at 96 hr from WM1158 cells that were not transfected (a), had received only Lipofectamine 2000 (b), or were transfected with 25 nM of control siRNAs (c) served as controls. For loading control, the immunoblot was probed with an antibody to α-tubulin. (**C**) WM1158 MGP melanoma cells, transfected for 48 hr with 75 nM of control siRNAs (a) or 75 nM of DDX11 siRNA (b) were analyzed by immunofluorescence-based TUNEL. Melanoma cells that had undergone apoptosis are pseudocolored red (TUNEL staining) and fluorescent DAPI-counterstained nuclei are pseudocolored blue.

To determine whether in addition to the observed defects in chromosome segregation, inhibition of DDX11 expression would result in interference with other biological features of melanoma cells, we transfected melanoma cells (1x10^5^/60 mm plate) with 25 nM of DDX11 or 25 nM of the pool of control siRNAs. Twenty-four hours after transfection, and every 24 hr thereafter, we determined the rate of cell proliferation. The results of this analysis, shown in Figure 4A, provided strong evidence that unlike melanoma cells that had received only Lipofectamine 2000 or were transfected with the control siRNAs, downregulation of DDX11 expression strongly inhibited the cells’ proliferation even as long as 96 hr post transfection. In light of the fact that DDX11 is an evolutionary highly conserved gene, it is of interest to note that in the absence of chl-1 (DDX11), C. elegans animals have been found to have proliferation defects in their soma and germline [12], and that in mammalian cells, DDX11 interacts with the proliferating cell nuclear antigen (PCNA) [9].

However, the most severe DDX11 siRNA-induced alteration we detected was manifest in the fact that the cells rapidly and massively underwent apoptosis. Depicted in Figure 4B, lanes d-g are the results of a c-PARP immunoblot analysis, which as reflected by cleavage of PARP to cPARP, documented that WM1158 MGP melanoma cells underwent apoptosis as early as 48 hr following transfection of the DDX11 siRNA (Figure 4B, lane e). In comparison, immunoblot analysis of WM1158 MGP melanoma cells that were not transfected (Figure 4B, lane a), had received only Lipofectamine 2000 (Figure 4B, lane b), or were transfected with 25 nM of the pool of control siRNAs (Figure 4B, lane c), did not demonstrate cleavage of PARP to cPARP even at 96 hr post transfection (Figure 4B, lanes a-c). A similar result was obtained by performing a TUNEL assay. Melanoma cells that as demonstrated in Figure 4C, panel a, had been transfected for 48 hr with 75 nM of the pool of four control siRNAs, did not reveal any TUNEL staining, but melanoma cells that had received the DDX11 siRNA clearly exhibited apoptosis as reflected by strong TUNEL staining (Figure 4C, panel b).

## Discussion

The findings of the study presented herein provide evidence that the DEAD/H (Asp-Glu-Ala-Asp/His) box helicase, DDX11, is upregulated with progression from early to advanced melanoma, and that this gene plays a pivotal role in shielding advanced melanomas from chromosome segregation defects and apoptosis.

Overshadowed for many years by efforts to find efficacious immunotherapies for advanced melanoma, and more recently by the focus on molecular therapy to target BRAF-mutated melanomas, considerably less attention has been paid to genes that are not only upregulated to high levels in advanced melanoma, but also play a pertinent role in protecting VGP and MGP melanomas from apoptosis. In a recent study [13], we documented by way of molecular targeting of the cell cycle regulator, CDK2, that VGP and MGP melanoma cells are highly vulnerable to interference with their progression through S phase. The data, summarized herein, provide not only further and strong support for this observation, but also demonstrate that inhibiting expression of a helicase such as DDX11, which has a vital function in sister chromatid cohesion [7,9,11], is deleterious for melanoma cells.

Thus far, little is known regarding the involvement, function, and importance of helicases in the progression from early to advanced melanoma, and their role in locally advanced and/or stage IV melanoma. Melanoma differentiation antigen 5 (MDA5), which comprises a caspase activation and recruitment domain (CARD) and an RNA helicase domain with ATP-dependent RNA-unwinding activity, was first isolated from a melanoma cell line [14]. However, induced by Interferon-β (IFN-β), MDA5 is not expressed in cells representing advanced melanoma unless the cells are treated with the cytokine. A second helicase, recently shown to be expressed in a subpopulation of melanoma cells, referred to as ABCB5+ malignant melanoma-initiating cells, is HAGE (alias Cancer/testis antigen 13 (CT13); DDX43) [15]. HAGE was shown to promote proliferation and tumor growth of this subpopulation of melanoma cells, and to regulate AKT and ERK phosphorylation through NRAS [15].

The novel and important findings regarding the herein described pivotal role of DDX11 in advanced melanoma is that following inhibition of DDX11 expression, the cells not only exhibited a significantly higher number of chromosomes with partially closed as well as open/separated arms, but also that compared with the control, the average length of their chromosomes was shorter. To date, little if anything is known about how VGP and MGP melanoma cells guard against DNA damage, control and maintain their genome stability, and related to these survival processes, retain telomere length. In the context of a study published a few years ago [16], a hypothesis was put forward but not tested that DDX11 might be involved in telomere length determination. Recently, however, it has been documented that loss of DDX11 leads to changes in telomeric chromatin formation [17], and that DDX11 interacts with the flap structure-specific endonuclease 1 (FEN-1) gene [9], which has a vital role in telomere stability. Thus, it is possible that like DDX39, which when overexpressed leads to progressive telomere elongation and to telomere shortening when depleted [18], DDX11 has an important function in maintaining telomere length and stability in a malignancy such as advanced melanoma. The second and even more pertinent finding described herein is that inhibition of DDX11 expression leads to rapid and massive melanoma cell apoptosis. In the context of mouse mutant studies, it has been shown that loss of Ddx11 causes apoptosis [11,19], but this is the first study which shows that inhibiting DDX11 expression in a malignancy that is refractory to virtually all apoptosis-inducing agents/therapies, leads to rapid and massive programmed cell death.

The biochemical functions of DDX11 have been established [20], but hitherto, a DDX11-specific small-molecule inhibitor is not available that would make it possible to systemically treat human melanoma xenografts and establish in vivo, therapeutic efficacy of blocking the function of DDX11. Given the possibility that like in the case of the Werner syndrome helicase [21], a human DDX11-specific small-molecule inhibitor will be isolated in the near future, it will be of importance to determine whether systemic therapy with such an inhibitor, alone or in combination with an inhibitor that blocks the function of FGFR1, which together with bFGF (FGF2) is a key regulator of melanoma proliferation, will have efficacy for advanced melanomas, and in particular for melanomas that are BRAF wild-type, which are the most aggressive type of advanced melanoma. Another interesting and important aspect that begs exploration is whether there is a link between DDX11 and pigmentation. Genetic disorders such as Bloom syndrome, Fanconi anemia, and the recently identified cohesinopathy, Warsaw breakage syndrome [8], are associated with abnormal skin pigmentation. Thus, it is a possibility that there is link between helicases such as DDX11 and the microphthalmia-associated transcription factor (MITF), which is a master regulator of melanocyte development.

In addition to the findings of our study presented herein, novel and compelling evidence that some members of the family of DEAD box helicases have pertinent functions in certain types of cancer has recently also been obtained in two other cases – one being breast cancer and the other medulloblastoma, which like melanoma is a neural crest-derived malignancy. In the case of breast cancer, the DEAD box helicase, DDX5, was found to be amplified and often co-amplified along with ERBB2, and breast cancer cell lines with amplification of the DDX5 locus were considerably more sensitive to its knockdown than breast cancer cell lines lacking this amplification [22]. In the case of WNT-subgroup medulloblastomas that unlike the other three medulloblastoma subtypes have a good long-term prognosis, exome sequencing revealed somatic missense mutations in the gene DDX3X [23-25], which is a paralogue of the DEAD box helicase, DDX3.

## Conclusions

The novel finding presented herein documents that DDX11, a member of the DEAD-box family of helicases, is expressed at high levels in primary and metastatic melanoma, but not in melanocytes of normal skin. Furthermore, our data demonstrate that interfering with the expression of DDX11 has severe consequences for melanoma cells. In particular, we document that inhibiting DDX11 expression causes substantial chromosome segregation defects and telomere shortening, major inhibition of melanoma cell proliferation, and rapid and massive melanoma cell apoptosis. Taken together, these data suggest that molecular targeting of DDX11 could be a new avenue and powerful approach to treat advanced melanoma, which is refractory to chemotherapy and radiation therapy.

## Methods

### Melanoma cell lines and tissues

VGP (WM983-A) and MGP (WM983-B, WM852, WM1158) human melanoma cell lines were propagated in vitro as previously described [26]. Standard immunohistochemistry analysis of deidentified, post-diagnosis excess cryopreserved 20 human tissue samples (deemed exempted (4e) by the University of Pittsburgh IRB), representing normal skin, atypical nevus, melanoma in situ, VGP melanoma, MGP melanoma, and melanoma-infiltrated lymph nodes was performed with an anti-human DDX11 mouse monoclonal antibody (Sigma-Aldrich, St. Louis, MO, USA). The chromogen used in the immunohistochemistry analysis was Vulcan Fast Red, and all DDX11 antibody-probed tissue sections were counterstained with hematoxylin.

### Immunoblot and immunofluorescence analysis

Protein lysates (25 μg/sample), separated on sodium dodecyl sulfate-polyacrylamide gels (SDS-PAGE) and transferred onto nylon membrane, were probed with antibody to human DDX11 (Sigma-Aldrich) or α-tubulin (Cell Signaling Technology, Inc., Danvers, MA, USA), followed by incubation with a horseradish peroxidase-conjugated secondary antibody (Cell Signaling Technology) and Luminol reagent (Millipore, Billerica, MA, USA).

For immunofluorescence analysis, melanoma cells were fixed with 2% paraformaldehyde (2 min at room temperature) and thereafter, with methanol (30 min at −20°C). The fixed cells were subsequently blocked with goat serum, probed with primary antibody to human DDX11 (Sigma-Aldrich) followed by an Alexa Fluor 555-conjugated secondary antibody (Invitrogen, Carlsbad, CA, USA), counterstained with fluorescent 40-6-diamidino-2-phenylindole (DAPI) (Invitrogen), and imaged with an inverted, epifluorescent TE2000 Nikon microscope and a charge-coupled device (CCD) camera (Roper Scientific, Inc./Photometrics, Tucson, AZ, USA).

### DDX11 siRNA conjugation and transfection

Five μg of a custom-synthesized siRNA (Thermo Fisher Scientific Dharmacon (Lafayette, CO, USA) based upon the human DDX11 exon 3-specific sequence 5’ CCU GUG UCU GUC UUC UUC CUG CGA A 3’ [10], which as we determined via a BLAST search, does not align with any other sequence, was conjugated to the fluorochrome Cy5 via a Label IT siRNA Tracker Intracellular Localization Cy5 kit (Mirus Bio LLC, Madison, Wl, USA). Melanoma cells, transfected for 24 hr with 5 nM of the Cy5-conjugated DDX11 siRNA, were fixed with 4% paraformaldehyde, counterstained with fluorescent DAPI (Invitrogen), and imaged. Dual color images were processed with MetaMorph software 7.7.5.0 (Molecular Devices, LLC, Sunnyvale, CA, USA).

Melanoma cells were transfected with the single DDX11 siRNA not conjugated or a control ON-TARGETplus non-targeting siRNA pool using Lipofectamine 2000 (Invitrogen) as the siRNA delivery vehicle. Phase-contrast images of the transfected melanoma cells were acquired with an inverted, epifluorescent TE2000 Nikon microscope and a CCD camera (Roper Scientific, Inc./Photometrics).

### qPCR analysis

Total RNA was isolated from DDX11 and control siRNA transfected melanoma cells with Trizol reagent (Sigma-Aldrich) and in each case, cDNA was transcribed with qscript cDNA Supermix (Quanta BioSciences, Inc., Gaithersburg, MD, USA) from 1 μg of RNA. qPCR reactions were performed with a pair of qPCR primers (life technologies/Applied Biosystems, Foster City, CA, USA) spanning human DDX11 exon boundary 20–22 that generated an 88 bp amplicon. A set of human 18s RNA primers (life technologies/Applied Biosystems) served as the internal control. Using PerfeCTa FastMix II, ROX (Quanta BioSciences, Inc.), 40 qPCR cycles (3 min at 95°C, 15 seconds at 95°C, 1 min at 95°C) were carried out via a StepOnePlus Real-Time PCR system (life technologies/Applied Biosystems). The qPCR data were analyzed using the 2^-ΔΔC^_T_ method.

### Chromosome staining and analysis

Following siRNA transfection, melanoma cells were treated for 90 min with 100 ng/ml of colchicine, followed by addition of hypotonic buffer (0.075 M KCl) for 15 min at 37°C and subsequent fixation with Carnoy's solution. The chromosome spreads were stained with fluorescent DAPI or Giemsa. Images of the fluorescent DAPI-stained chromosome spreads were captured with an inverted, epifluorescent TE2000 Nikon microscope and a CCD camera (Roper Scientific, Inc./Photometrics). Images of the Giemsa-stained chromosome spreads were acquired with a Hamamatsu NanoZoomer 2.0-HT (Hamamatsu Corporation USA, Bridgewater, NJ, USA). The number of chromosomes with closed, partially closed, or open/separated arms was determined for 40 Giemsa-stained chromosome spreads. The ‘Linear Measure’ tool in the NDP.view software (Hamamatsu Corporation) was used to determine the length of three randomly selected chromosomes in each of 50 Giemsa-stained chromosome spreads.

### Proliferation and apoptosis analysis

Melanoma cell proliferation was determined by counting cells with a hemocytometer. At each time point, duplicate samples were analyzed for DDX11 as well as control siRNA-transfected cells, and cells that had received only Lipofectamine 2000 (Invitrogen).

Whole-cell lysates (30 μg/sample) of melanoma cells transfected with DDX11 or control siRNA were separated by SDS-PAGE, transferred onto nylon membrane, and probed with antibody to c-PARP (Cell Signaling Technology, Inc.) or α-tubulin (Cell Signaling Technology, Inc.), followed by incubation with a horseradish peroxidase-conjugated secondary antibody (Cell Signaling Technology) and Luminol reagent (Millipore).

For immunofluorescence-based detection of apoptosis, cytospin preparations of DDX11 as well as control siRNA-transfected melanoma cells were fixed, permeablized, labeled with the In Situ Cell Death Detection Kit, TMR red (Roche Applied Science, Indianapolis, IN, USA), counterstained with fluorescent DAPI, and imaged.

## Abbreviations

qPCR: Quantitative polymerase chain reaction; Giemsa: Solution for staining chromosomes; BRAF: v-raf murine sarcoma viral oncogene homolog B1; CTLA-4: Cytotoxic T-Lymphocyte Antigen 4; DASL: cDNA-mediated Annealing, Selection, Extension, and Ligation assay; FANCJ: Fanconi anemia complementation group J; XPD: Xeroderma pigmentosum, complementation group D; RTEL: Regulator of telomere elongation helicase 52; PARP: Poly ADP (Adenosine Diphosphate)-Ribose Polymerase; TUNEL: Terminal Uridine Nucleotide End-Labeling; CDK2: Cyclin-dependent kinase 2; ATP: Adenosine triphosphate; AKT: Protein kinase B; ERK: Extracellular signal-regulated kinase; NRAS: Neuroblastoma RAS Viral Oncogene Homolog; FGFR1: Fibroblast growth factor receptor 1; bFGF (FGF2): Basic fibroblast growth factor (fibroblast growth factor 2); ERBB2: v-erb-b2 erythroblastic leukemia viral oncogene homolog 2; WNT: Combination of Wg (wingless) and Int; BLAST: Basic Local Alignment Search Tool.

## Competing interests

The authors declare that they have no competing interests.

## Authors’ contributions

CB performed the studies that involved siRNA transfection, qPCR, preparation and analysis of chromosome spreads, and proliferation and apoptosis assays. CB also helped to draft the manuscript. XW carried out the immunohistochemistry study, and the immunoblot and optical imaging analysis. DB conceived of the study, participated in its design and coordination, and wrote the manuscript. All authors read and approved the final manuscript.
